# CoenzymeQ10-Induced Activation of AMPK-YAP-OPA1 Pathway Alleviates Atherosclerosis by Improving Mitochondrial Function, Inhibiting Oxidative Stress and Promoting Energy Metabolism

**DOI:** 10.3389/fphar.2020.01034

**Published:** 2020-07-22

**Authors:** Tianqi Xie, Changyuan Wang, Yue Jin, Qiang Meng, Qi Liu, Jingjing Wu, Huijun Sun

**Affiliations:** ^1^ Department of Clinical Pharmacology, College of Pharmacy, Dalian Medical University, Dalian, China; ^2^ Academy of Integrative Medicine, Dalian Medical University, Dalian, China

**Keywords:** coenzyme Q10, atherosclerosis, oxidative stress, energy metabolism, AMPK-YAP-OPA1

## Abstract

Atherosclerosis (AS) is an excessive chronic inflammatory hyperplasia caused by the damage of vascular endothelial cell morphology and function. Changes in mitochondrial internal conformation and increase of reactive oxygen species (ROS) can lead to energy metabolism disorders in mitochondria, which further affects the occurrence of atherosclerosis by impairing vascular endothelial function. Coenzyme Q10 (CoQ10) is one of the components of mitochondrial respiratory chain, which has the functions of electron transfer, reducing oxidative stress damage, improving mitochondrial function and promoting energy metabolism. The main purpose of this study is to investigate the protective effects of CoQ10 against AS by improving mitochondrial energy metabolism. Both in high fat diet (HFD) fed APOE**^−/−^** mice and in ox-LDL-treated HAECs, CoQ10 significantly decreased the levels of TG, TC and LDL-C and increased the levels of HDL-C, thus playing a role in regulating lipid homeostasis. Meanwhile, CoQ10 decreased the levels of LDH and MDA and increased the levels of SOD and GSH, thus playing a role in regulating oxidation level. CoQ10 also inhibited the over-release of ROS and increased ATP content to improve mitochondrial function. CoQ10 also decreased the levels of related inflammatory factors (ICAM-1, VCAM-1, IL-6, TNF-α and NLRP3). In order to study the mechanism of the experiment, AMPK and YAP were silenced *in vitro*. The further study suggested AMPK small interfering RNA (siRNA) and YAP small interfering RNA (siRNA) affected the expression of OPA1, a crucial protein regulating the balance of mitochondrial fusion and division and decreased the therapeutic effects of CoQ10. These results indicated that CoQ10 improved mitochondrial function, inhibited ROS production, promoted energy metabolism and attenuated AS by activating AMPK-YAP-OPA1 pathway. This study provides a possible new mechanism for CoQ10 in the treatment of AS and may bring a new hope for the prevention and treatment of AS in the future.

## Introduction

With the aging of population in the contemporary world, diseases of aging are getting more and more attention. The occurrence of atherosclerosis is closely related to a variety of chronic senile diseases, including coronary heart disease, cerebral infarction, peripheral vascular disease and so on. There are many theories about the pathogenesis of atherosclerosis (Su et al., 2019; Sun et al., 2019; Tsujikawa et al., 2019). In recent years, many reports suggest that atherosclerosis is closely associated with energy metabolism disorder (Antoniades et al., 2009; Yu et al., 2017). Mitochondrial energy metabolism disorder can produce a large number of ROS, which can accelerate the occurrence of inflammation and lead to damage to vascular endothelial function. Meanwhile, in the process of atherosclerosis, the disorder of mitochondrial function can lead to the decrease of electron transport chain load, membrane potential, DNA damage, abnormal energy metabolism and reduced ATP production. Indeed, atherosclerosis and mitochondrial energy metabolism disorder are cause-and-effect in atherosclerosis (Chiong et al., 2014; Pircher et al., 2016; Dong et al., 2017; Vanhoutte et al., 2017). However, the specific underlying mechanism is not yet clear. Thus, finding a drug that plays an important role in improving disorder of energy metabolism and exploring its mechanism of prevention and treatment of atherosclerosis is of great significance for further study and treatment of atherosclerosis.

CoQ10, also known as ubiquinone, is a lipid-soluble quinone compound existing in nature. Ubiquinone are present in most eukaryotic cells, especially in mitochondria. Recently, pharmacological studies have shown that CoQ10, as an essential component of the respiratory chain, is an electron transmitter in the electron transport chain. It can scavenge free radicals, reduce oxidative stress damage, improve mitochondrial function, promote oxidative phosphorylation process, and produce ATP to promote energy metabolism (Perez-Sanchez et al., 2012; Morris et al., 2013; Jing et al., 2015; Fatima et al., 2016). At the same time, CoQ10 can also inhibit inflammation and improve HDL function, thus playing a positive role in inhibiting atherosclerosis formation and reducing plaque rupture (Wang D. et al., 2014; Li et al., 2017). At present, there are many reports of CoQ10 on the prevention and treatment of atherosclerosis but few on regulating mitochondrial energy metabolism by CoQ10, and the potential mechanism and drug targets are fairly unknown (Gairola et al., 2010; Gao et al., 2012; Tsai et al., 2012; Allen and Vickers, 2014).

A large number of literatures have reported that oxidized low-density lipoprotein can damage vascular endothelial cells and promote the formation of foam cells, which is an important cause of atherosclerosis. Therefore, *in vitro* experiments were conducted to investigate the effects of CoQ10 on oxidative stress, inflammation, mitochondrial function and energy metabolism in ox-LDL-induced HAEC cells, and to explore its mechanism.

Adenosine 5’-monophosphate (AMP)-activated protein kinase(AMPK)is a key enzyme involved in cell energy metabolism and can be used as an energy sensor of cells. The role of AMPK in inhibiting oxidative stress and preventing endothelial dysfunction has been confirmed by many studies (Chen et al., 2013; Fan et al., 2015; Yang et al., 2016). AMPK not only inhibits inflammation, but also promotes oxidative phosphorylation and ATP production (Kahn et al., 2005; Colombo and Moncada, 2009; Ke et al., 2018). Therefore, we suspect that AMPK may be involved in the prevention and treatment of atherosclerosis through energy-saving metabolism (Zong et al., 2002; Chen K. et al., 2019; Katsiougiannis et al., 2019). Hippo pathway is a kinase chain composed of a series of protein kinases and transcription factors. Previous studies have suggested that Hippo pathway is involved in cell survival, proliferation, regeneration and other processes, and plays an key role in regulating the size of organs and tissue homeostasis (Adler et al., 2013a; Fan et al., 2013; Taniguchi et al., 2015). Moreover, Hippo pathway can participate in metabolic processes at the cellular level and play an important regulatory role in metabolic diseases such as non-alcoholic fatty liver disease, type 2 diabetes mellitus, myocardial disorders and cancer (DeRan et al., 2014; Wang et al., 2015). Accumulating evidences have proved that activation of AMPK can promote the phosphorylation of YAP (Nagaraj et al., 2012; Wang et al., 2012; Adler, 2013b; Wang Y. et al., 2014; Choi et al., 2015), an important effector molecule in the Hippo pathway, so that YAP in the cytoplasm cannot enter the nucleus to exert transcriptional activity and maintain energy homeostasis through negative regulation of YAP in metabolic-related pathways (Sorrentino et al., 2014; Ardestani et al., 2018; Wu et al., 2019). OPA1 (Optic Atrophy 1) gene belongs to nuclear gene. OPA1 is located in the mitochondrial inner membrane and is a crucial factor regulating the balance of mitochondrial fusion and division. As a part of respiratory chain, OPA1 can maintain the integrity of respiratory chain, the morphology and function of mitochondria, OPA1 also participates in respiratory and energy metabolism (Guillery et al., 2008; Merkwirth et al., 2008; Loucks et al., 2009; Suárez-Rivero et al., 2018; Chen X. et al., 2019). At present, AMPK–YAP–OPA1 pathway has been reported in the treatment of renal ischemia–reperfusion injury, cerebral ischemia–reperfusion injury, cardiac reperfusion stress and other diseases, but there is no report on the treatment of atherosclerosis (Deng et al., 2016; Wang et al., 2016; Sun et al., 2017; Zhang et al., 2019). Thus, we speculate that CoQ10 may prevent and treat atherosclerosis through AMPK–YAP–OPA1 pathway.

## Materials and Methods

### Reagents

CoQ10 (98%) was obtained from Nanjing Jingzhu Biotech Ltd. Co. (Nanjing, Jiangsu, China). The DMEM culture medium was bought from Gibco-BRL Company (Gaithersburg, MD, USA). Antibodies specific for IL-6,TNF-α, ICAM-1, VCAM-1, NLRP3, p-AMPK,AMPK,YAP and β-actin were obtained from Proteintech Group (Wuhan, Hubei, China). Antibodies specific for p-YAP and OPA-1 were bought from Abcam (Cambridge, MA, USA).

### Preparation of ox-LDL

The LDL was obtained from the normal plasma by gradient ultracentrifugation with Beckman Coulter Optima L-100 XP Ultracentrifuge. The LDL was oxidized by 50 μM CuSO4 at 37°C dark for 24 h. The oxidized LDL was put into phosphate-buffered saline (PBS) containing 200mmol/L EDTA for dialysis for 24 h. Then, it was further dialyzed with 0.01% EDTA and filtered for standby. MDA kit was used to test whether the oxidized LDL reached the oxidation standard and BCA kit was used to detect concentration. LDL was prepared every 14 days.

### Ethics Statement

All the procedures were performed in compliance with the Institute’s guidelines and the Guide for the Care and Use of Laboratory Animals. The study was approved by the institutional animal care committee of Dalian Medical University.

### Animals and Diets

Male eight-week-old apolipoprotein E genes knockout (APOE**^−/−^**) mice were purchased from Vital River Laboratory Animal Technology Co., Ltd. (Beijing, China). C57BL/6 mice were bought from Liaoning Changsheng biotechnology Co., Ltd. (Benxi, China). Before the experiment was begun, mice were exposed at room temperature, kept the conditions of light and dark circulation, and were given normal diet. After a week, the mice were sufficiently adapted to the new environment. To explore the effect of CoQ10 on atherosclerosis *in vivo*, we divided the mice into the following groups. Normal diet group (n = 10), simple treatment group (CoQ10 100 mg/kg/D, n = 10), model group (HFD, n = 10), low dose treatment group (HFD, CoQ10 100 mg/kg/D, n = 10), high dose treatment group (HFD, CoQ10 200 mg/kg/D, n = 10). Normal diet group and model group were given peanut oil by gavage every day. Simple treatment group, low dose treatment group and high dose treatment group were given CoQ10 (dissolved in peanut oil) by gavage daily for 12 weeks. At the end of the experiment, blood was collected from the eyeballs of mice and aortic tissue was taken out for analysis.

### Mice Body Weight

In order to observe the weight change of mice, the weight of mice was measured and recorded every two weeks within 12 weeks.

### Detection of Histopathological Changes by Oil Red O Staining

The abdominal aortas of five groups of mice were stained. The tissues were washed with distilled water and 60% isopropanol and stained with oil red O. Then, the tissues were fixated with 4% paraformaldehyde and observed the pathological changes in photos under stereomicroscope.

### HE Staining of Tissue Sections

The aorta was fixed in formalin solution and cut into cross sections. The aorta was dehydrated overnight with 75% ethanol and then embedded in paraffin. The sections were stained with hematoxylin and eosin for histologic analysis. All pathological slices were collected by Olympus Positive Microscope and analyzed by Image-pro Plus 6.0 (Media Cybernetics, Inc.).

### Cell Culture

Human aortic endothelial cells (HAECs) were purchased from Shanghai Bioleaf Biotech Co., Ltd. (Shanghai, China). Cells were cultured in DMEM medium containing 10% FBS. Meanwhile, cells grow in incubators containing 5% CO_2_ and at 37°C. Wash with PBS every other day and replace the culture medium. When the cell growth reached a confluence of 70–80%, it was digested by trypsinase and distributed into two culture flasks for further passage. Atherosclerosis model was received 24 h after culture with ox-LDL (150 μg/ml) instead of culture medium.

### Cell Viability Assay

HAECs were cultured in 96-well plates at a density of about 5–10 × 10^3^/ml. 96-well plates are placed in a 37 °C incubator containing 5% CO_2_ and the air inside the plates is kept moist. Twenty four hours later, replace the complete medium with blank medium and add different concentration of CoQ10 (10, 20, and 40 μM) with or without ox-LDL (150 μg/ml). Meanwhile, cells without any treatment were used as control group. Based on above, after 24 h, 5 mg/ml methyl thiazolyl tetrazolium (MTT) was added to every well for 4h. Then, absorbing the liquid from the hole and adding the triple liquid. Approximately 12–15 h later, measurement of OD at 570 nm by microplate reader (Thermo Fisher Scientific, MA, USA).

### Detection of Biochemical Indicators

Before the analysis, samples were collected from mice serum and cells for treatment and preservation. The levels of superoxide dismutase (SOD), glutathione (GSH), malondialdehyde (MDA), lactate dehydrogenase (LDH), total cholesterol (TC), triglyceride (TG), high density lipoprotein cholesterol (HDLC), low density lipoprotein cholesterol (LDLC) were measured by detection kits based on the manufacturer’s instructions (Nanjing Jiancheng Institute of Biotechnology, Nanjing, China) in serum and cells.

### Detection of Intracellular ROS

Intracellular ROS levels were measured using a 2’, 7’-dichlorofluorescent yellow diacetate (DCFH-DA) probe. HAECs were cultured in small petri dishes and treated with CoQ10 (0, 10, 20, 40 μM) and with or without ox-LDL (150 μg/ml) for 24 h. Then the cells were incubated for 30 min with 10mM DCFH-DA probe at 37 °C. After incubation, the cells were digested with trypsin, centrifuged and collected. Detection of ROS in collected cells by flow cytometry.

### JC-1 Staining

JC-1 staining was used to investigate the effect of coenzyme Q10 on mitochondrial membrane potential induced by ox-LDL in HAECs. HAECs were cultured in 6-well plates and treated with CoQ10 (0, 10, 20, 40 μM) and with or without ox-LDL (150 μg/ml) for 24 h. Absorbing the liquid in the hole, adding JC-1 dyeing working fluid (10 mg/ml), incubating in incubator at 37° for 20 min. Then, wash twice with JC-1 dyeing buffer and add culture medium. Observation of fluorescence changes under inverted microscope.

### Detection of ATP Content

Cellular ATP generation was measured to reflect mitochondrial function. First, cells were washed with cold PBS three times at room temperature. Then, ATP content assay kit (Nanjing Jiancheng Institute of Biotechnology, Nanjing, China) was used to analyze the ATP content according to the instructions. ATP production was measured *via* a microplate reader.

### Western Blotting

The protein extracts were extracted from mice blood vessels and HAECs. Proteins were separated in sodium dodecyl sulfate-polyacrylamide electrophoresis gels (8–15%) and then transferred to a PVDF membrane (Millipore, Bedford, MA, USA). The PVDF membranes containing the target protein was sealed with 5% skimmed milk for about 2 h. Then, incubate the first antibodies and stay overnight at 4 °C. After the PVDF membranes were cleaned with TTBS, the second antibodies were incubated. After the above operations are completed, PVDF membranes were in full contact with chemiluminescent reagents (Beyotime). The final images were captured by a Bio-Rad imaging system and analyzed with a Gel-Pro Analyzer Version 4.0 (Media Cybernetics, MD, USA).

### ELISA

ELISA assay kit was used to investigate the effect of CoQ10 on the level of IL-6 and TNF-α induced by ox-LDL in HAECs. HAECs were cultured in 6-well plates and treated with CoQ10 (0, 10, 20, 40 μM) and with or without ox-LDL (150 μg/ml) for 24 h. Following the manufacturer’s instructions., the IL-6 and TNF-α activities was recorded *via* a microplate reader to reflect.

### Transfection

Mate and si-AMPK, si-YAP sequence or negative control sequence were mixed into 1 ml serum-free DMEM medium, and then the mixture was added into each pore for 10 min. Cells transfected with NS or specific siRNA for 24 h were treated with ox-LDL or coenzyme Q10 for an additional 24 h, then harvested and analyzed by Western blotting analysis.

AMPK siRNA (sense: 5’-CCAUUCUUGGUUGCUGAAATT-3’; antisense: 5’-UUUCAGCAACCAAGAAUGGTT-3’)

YAP siRNA (sense: 5’-GACGACCAAUAGCUCAGAUTT-3’; antisense: 5’-AUCUGAGCUAUUGGUCGUCTT-3’)

Negative control (sense: 5’-UUCUCCGAACGUGUCACGUTT-3’; antisense: 5’-ACGUGACACGUUCGGAGAATT-3’)

### Statistical Analysis

All the results were analyzed by SPSS 17.0 and GraphPad Prism 5 software and they are expressed as the mean ± standard deviation (SD) from no less than three independent experiments. ANOVA (Analysis of Variance) or Student’s t-test were used to compare the differences between the results of each group. P values <0.05 (two-tailed) were considered statistically significant.

## Result

### Effects of CoQ10 on Body Weight, Aortic Lesions and Lipid Homeostasis in HFD-Induced APOE^−/−^ mice

**Figure 1:**
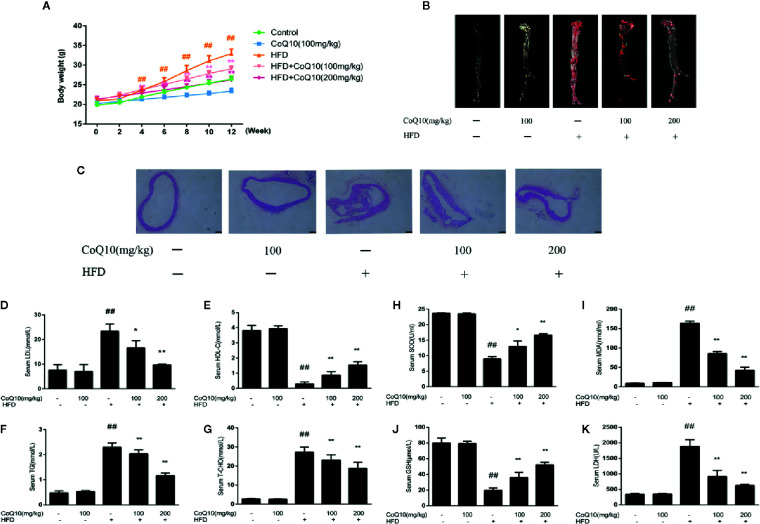
CoQ10 improved body weight, aortic lesions, lipid homeostasis and antioxidant capacity in HFD-induced APOE^−/−^ mice. **(A)** Weight change of mice. **(B)** Oil red O staining on aortic tissue of APOE**^−/−^** mice. **(C)** HE staining on aortic tissue of APOE**^−/−^** mice. **(D)** Serum level of LDL. **(E)** Serum level of HDL. **(F)** Serum level of TG. **(G)** Serum level of TC. **(H)** Serum level of SOD. **(I)** Serum level of MDA. **(J)** Serum level of GSH. **(K)** Serum level of LDH. Results are mean ± SD (n = 10). ^##^p < 0.01 *vs.* Control group, ^**^p < 0.01 *vs.* HFD group, ^*^p < 0.05 *vs.* HFD group.

To investigate the effect of CoQ10 on the body weight of APOE**^−/−^** mice fed with HFD, the body weight of the mice was recorded every two weeks. The result showed that the mice in HFD group are the heaviest. However, the weight gain of mice treated with CoQ10 was relatively slow (**Figure 1A**). Oil red O staining can be more intuitive to see the aortic lesions. As seen from the figure, the aortic plaque area in HFD group was larger and the plaque was much more obvious compared to the control group. After treatment with CoQ10, the atherosclerotic plaque decreased significantly compared to the HFD group (**Figure 1B**). HE staining indicated that the aortas from the HFD group exhibited an increased intimal lesion area containing a necrotic core. However, CoQ10 treatment could improve this condition (**Figure 1C**).

The serum levels of LDLc, HDLc, TG and TC were determined to investigate the effect of CoQ10 on lipid profiles. As shown in **Figures 1D, F, G**, serum LDLc, TG and TC levels in the HFD group were significantly increased. However, CoQ10 significantly decreased LDLc, TG and TC levels, especially in the high dose group. Conversely, HDLc levels were the lowest in the HFD group (**Figure 1E**), and CoQ10 reversed this phenomenon.

### CoQ10 Increased SOD and GSH Content and Decreased MDA Content and LDH Release in HFD-Induced APOE^−/−^ Mice

To detect the effect of CoQ10 on oxidative level in HFD-induced APOE**^−/−^** mice, serum SOD, GSH, MDA and LDH levels in APOE**^−/−^** mice were determined and the result showed (**Figures 1H, J**) that, SOD and GSH contents in HFD group were the lowest, indicating that the antioxidant capacity in HFD group was the weakest. After treatment with CoQ10, the contents of SOD and GSH were rescued gradually, and the antioxidant capacity improved. On the contrary, as shown in **Figures 1I, K** MDA and LDH levels were highest in HFD group, which were restored after treatment with CoQ10.

### Expression of AMPK, p-AMPK, YAP, p-YAP and OPA-1 Proteins in Blood Vessels of APOE^−/−^ Mice

**Figure 2:**
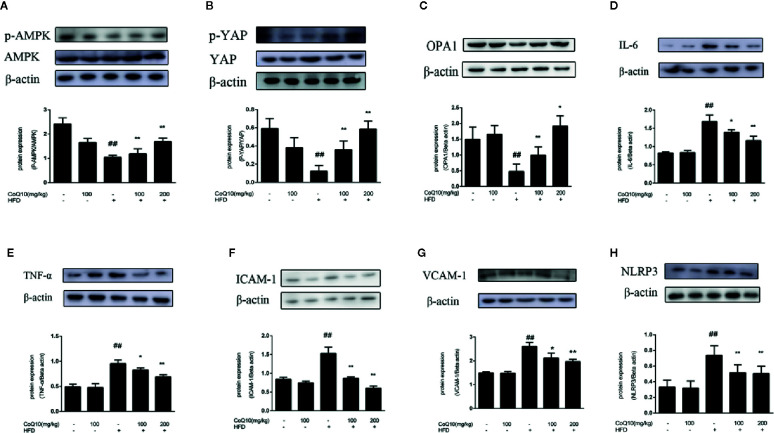
CoQ10 reversed down-regulation of phosphorylated AMPK and phosphorylated YAP, promoted OPA1 protein expression and alleviated vascular-associated inflammatory proteins induced by HFD-treated APOE^−/−^ mice. **(A)** phosphorylated AMPK and AMPK protein expression ratio. **(B)** phosphorylated YAP and YAP protein expression ratio. **(C)** OPA1 protein expression level. **(D)** IL-6 protein expression level. **(E)** TNF-α protein expression level. **(F)** ICAM-1 protein expression level. **(G)** VCAM-1protein expression level. **(H)** NLRP3 protein expression level. Results are mean ± SD (n = 10). ^##^p <0.01 *vs.* Control group, ^**^p <0.01 *vs.* HFD group, ^*^p <0.05 *vs.* HFD group.

To explore the role of CoQ10 in energy metabolism, the expression of related proteins was detected. As shown in **Figure 2A**, the ratio of phosphorylated AMPK to total AMPK in HFD group was the lowest, indicating that the effective expression of AMPK in HFD group was dramatically inhibited, and the ratio was markedly increased after treatment with CoQ10. However, the results of **Figure 2B** show that the ratio of phosphorylated YAP to YAP was obviously reduced in HFD group, while CoQ10 significantly enhanced the ratio, exhibiting promoting effect on the phosphorylation process of YAP. Then, the expression of OPA-1 protein was detected by western blotting. The results in **Figure 2C** showed that compared to the control group, OPA-1 protein expression was greatly depressed in the HFD group, suggesting that the mitochondrial function might be damaged. After treatment with CoQ10, the protein expression of OPA-1 was rescued. The above results illustrated that CoQ10 could increase the protein expression of AMPK, OPA-1and promote the phosphorylation of YAP, suggesting that CoQ10 might improve the mitochondrial function.

### Expression of Vascular-Associated Inflammatory Protein in APOE^−/−^ Mice

To further explore the effect of CoQ10 on inflammation in HFD-induced APOE**^−/−^** mice, the expression of IL-6, TNF-α, VCAM-1 and ICAM-1 proteins was detected. **Figures 2D–H** showed that the expression of IL-6, TNF-α, ICAM-1, VCAM-1 and NLRP3 in the HFD group is the most. Whereas CoQ10 could reverse this phenomenon, which indicated that CoQ10 could significantly attenuate inflammation in blood vessels.

### Effect of CoQ10 on the Cell Survival Rate in ox-LDL-Induced HAECs

**Figure 3:**
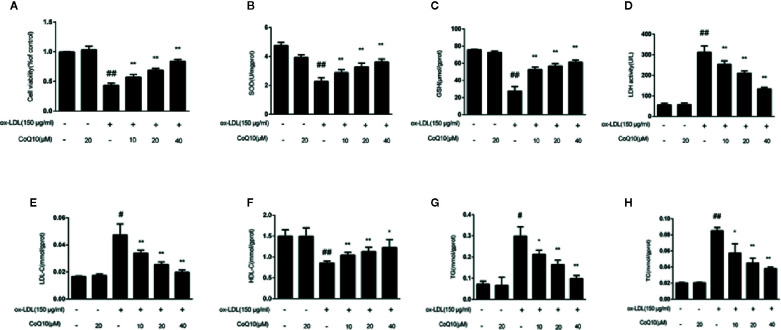
Effect of CoQ10 on cell viability, oxidation index and lipid homeostasis in ox-LDL-treated HAECs. **(A)** The cell viability. **(B)** The level of SOD. **(C)** The level of GSH. **(D)** The level of LDH. **(E)** The level of LDL. **(F)** The level of HDL. **(G)** The level of TG. **(H)** The level of TC. Data in figure represent the mean ± SD from three independent experiments. ^##^p < 0.01 *vs.* Control group, ^#^p < 0.05 vs. Control group, ^**^p < 0.01 *vs.* ox-LDL group, ^*^p < 0.05 *vs.* ox-LDL group.

In order to explore the protective effect of CoQ10 on ox-LDL induced HAECs, the cell viability was detected by MTT experiments in HAECs. The results of **Figure 3A** demonstrated that when ox-LDL concentration is 150 μg/ml, the cell viability was down reduced to 50%. However, treatment with different concentrations of CoQ10 (10, 20 and 40 μM), the cell viability was increased in a dose-dependent manner.

### Effect of CoQ10 on Blood Lipid Index in ox-LDL-Induced HAECs

The contents of TG, TC, LDL-c and HDL-c reflect the level of lipid homeostasis. The contents of TG, TC and LDL-c were the most in ox-LDL-induced group (**Figures 3E, G, H**), while the HDL-c (**Figure 3F**) was the least. CoQ10 could regulate lipid homeostasis in ox-LDL-induced HAECs.

### Effect of CoQ10 on Oxidation Index in ox-LDL-Induced HAECs

The contents of SOD, GSH and LDH reflect the capacity of antioxidant. **Figures 3B–D** demonstrated that CoQ10 could inhibit LDH release, but increase the contents of SOD and GSH in ox-LDL-induced HAECs, showing that CoQ10 could produce antioxidant effect *in vitro*.

### Effect of CoQ10 on Expression of AMPK, p-AMPK, YAP, p-YAP and OPA-1 Proteins in ox-LDL-Induced HAECs

**Figure 4:**
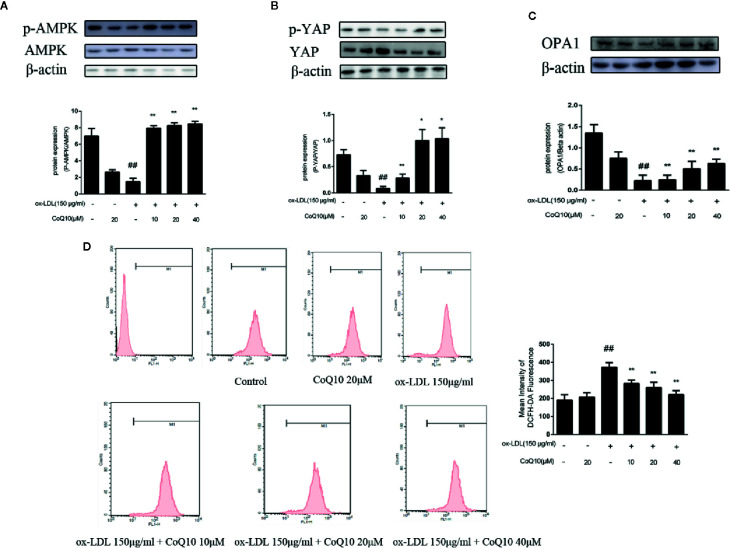
CoQ10 reversed down-regulation of phosphorylated AMPK and phosphorylated YAP, promoted OPA1 protein expression and decreased ROS generation in ox-LDL-treated HAECs. **(A)** phosphorylated AMPK and AMPK protein expression ratio. **(B)** phosphorylated YAP and YAP protein expression ratio. **(C)** OPA1 protein expression level. **(D)** The level of ROS. Data in figure represent the mean ± SD from three independent experiments. ^##^p < 0.01 *vs.* Control group, ^**^p < 0.01 *vs.* ox-LDL group, ^*^p < 0.05 *vs.* ox-LDL group.

As shown in **Figure 4A**, the ratio of phosphorylated AMPK to total AMPK in the ox-LDL-induced group was the lowest, indicating that the effective expression of AMPK was inhibited after exposure to ox-LDL, and the ratio increased significantly after treatment with CoQ10. Moreover, the results of **Figure 4B** showed that the ratio of phosphorylated YAP to YAP was also the lowest in the ox-LDL-induced group, while CoQ10 could promote the phosphorylation process of YAP and increase the ratio significantly. Then, the expression of OPA-1 protein was detected. The results in **Figure 4C** showed that OPA-1 protein expression was greatly down-regulated in the ox-LDL-induced group, indicating that mitochondrial function might be damaged under the action of ox-LDL. After treatment with CoQ10, the content of OPA-1 was rescued, suggesting that mitochondrial function may be improved in ox-LDL-induced HAECs.

### Effect of CoQ10 on ROS Production in ox-LDL-Induced HAECs

The content of ROS can reflect the oxidative stress in HAECs. The results of flow cytometry in **Figure 4D** demonstrated that the fluorescence intensity of cells in the ox-LDL-induced group was the strongest, suggesting that ox-LDL could result in the over-generation of ROS. And after treatment with CoQ10, the fluorescence intensity decreased in a dose-dependent manner, suggesting that CoQ10 could inhibit oxidative stress by inhibiting the over-generation of ROS in ox-LDL-induced HAECs.

### Effect of CoQ10 on Mitochondrial Membrane Potential in ox-LDL-Induced HAECs

**Figure 5:**
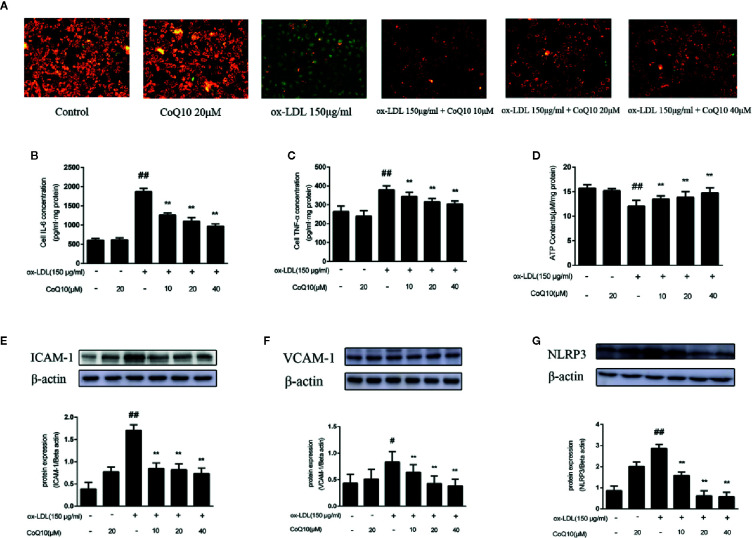
Coenzyme Q10 improved mitochondrial potential, increased ATP generation and alleviated vascular-associated inflammatory factors in ox-LDL-treated HAECs. **(A)** The JC-1 staining. **(B)** ELISA kit results of IL-6. **(C)** ELISA Kit results of TNF-α. **(D)** The level of ATP. **(E)** ICAM-1 protein expression level. **(F)** VCAM-1 protein expression level. **(G)** NLRP3 protein expression level. Data in figure represent the mean ± SD from three independent experiments. ^##^p < 0.01 *vs.* Control group, ^#^p < 0.05 *vs.* Control group, ^**^p < 0.01 *vs.* ox-LDL group.

JC-1 staining could reflect the changes of mitochondrial membrane potential. We found that the green fluorescence was enhanced in the ox-LDL-induced cells compared to the control cells and the green fluorescence gradually turned to red fluorescence after treatment with CoQ10 (**Figure 5A**).This result indicated that ox-LDL caused damage to the mitochondria of HAECs, resulting in the decrease of mitochondrial membrane potential and the decreased JC-1 dye uptake. However, CoQ10 could improve the mitochondrial function in ox-LDL-induced HAECs in a dose-dependent manner.

### Effect of Coenzyme Q10 on the ATP Content in ox-LDL-Induced HAECs

ATP content could reflect the energy metabolism of mitochondria, therefore we then determine ATP content in HAECs. From the results we could observed that the ATP content was mostly decreased in the ox-LDL-induced HAECs. In contrast, after treatment with CoQ10, the ATP content could be restored in a dose-dependent manner (**Figure 5D**), suggesting that CoQ10 could promote energy metabolism in ox-LDL-induced HAECs.

### Effect of CoQ10 on Related Inflammatory Factors in ox-LDL-Induced HAECs

The levels of IL-6 and TNF-α in HAECs were detected by ELISA kit. The results in **Figures 5B, C** showed that the highest levels of IL-6 and TNF-α were found in ox-LDL-induced HAECs, which could be reversed after treatment with CoQ10. Then, the expression of ICAM-1, VCAM-1 and NLRP3 in HAECs was detected, and the results were consistent with those *in vivo* (**Figures 5E–G**). It suggested that CoQ10 could inhibit the occurrence of inflammation in atherosclerosis.

### CoQ10 Promoted YAP Phosphorylation, OPA1 Expression and ATP Production in ox-LDL-Induced HAECs by Activating AMPK Pathway

**Figure 6:**
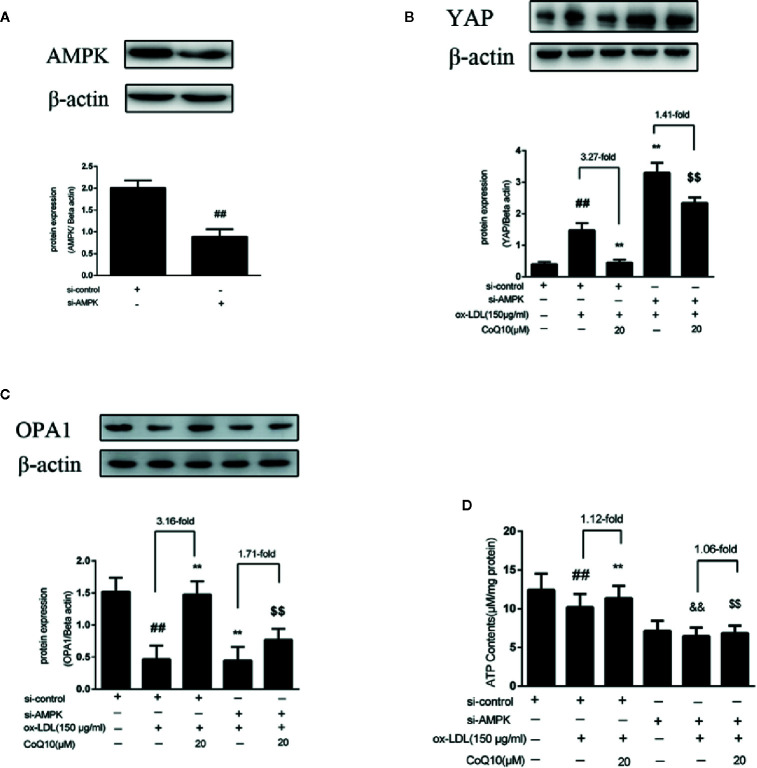
CoQ10 promoted YAP phosphorylation, OPA1 expression and ATP production in ox-LDL-induced HAECs by activating AMPK pathway. **(A)** AMPK protein expression level after transfection of AMPK si-RNA. **(B)** YAP protein expression level after si-AMPK. **(C)** OPA1 protein expression level after si-AMPK. **(D)** ATP content after si-AMPK. Data in figure represent the mean ± SD from three independent experiments. ^##^p < 0.01 *vs.* si-Control group, ^**^p < 0.01 *vs.* si-Control + ox-LDL, ^&&^p < 0.01 *vs.* si-AMPKgroup, ^$$^p < 0.01 *vs.* si-AMPK + ox-LDL group.

In order to further explore the experimental mechanism, AMPK was silenced. As observed from **Figure 6A**, AMPK was silenced successfully. Then, the expressions of YAP, OPA1 and the level of ATP after silencing AMPK were measured. The results showed that after AMPK knockdown, the overall trend of YAP expression was increased, and the effect of CoQ10 reducing YAP expression was greatly attenuated compared to before AMPK knockdown (**Figure 6B**).This indicated that AMPK could promote the phosphorylation of YAP lead to YAP decreased. Furthermore, **Figure 6C** showed that the expression of OPA1 was decreased after AMPK knockdown, and the effect of CoQ10 increasing OPA1 expression was weakened obviously compared to before AMPK knockdown. Moreover, **Figure 6D** showed that the overall trend of ATP content was decreased after AMPK knockdown, and the effect of CoQ10 elevating ATP content was also weakened. The above results suggested that the preventive effects of CoQ10 against atherosclerosis might be achieved by improving mitochondrial function and promoting energy metabolism through AMPK-YAP-OPA1 pathway.

### CoQ10 Inhibited Oxidative Stress, ROS Over-Production and Expression of Inflammatory Related Factors in ox-LDL-Induced HAECs by Activating AMPK Pathway

**Figure 7:**
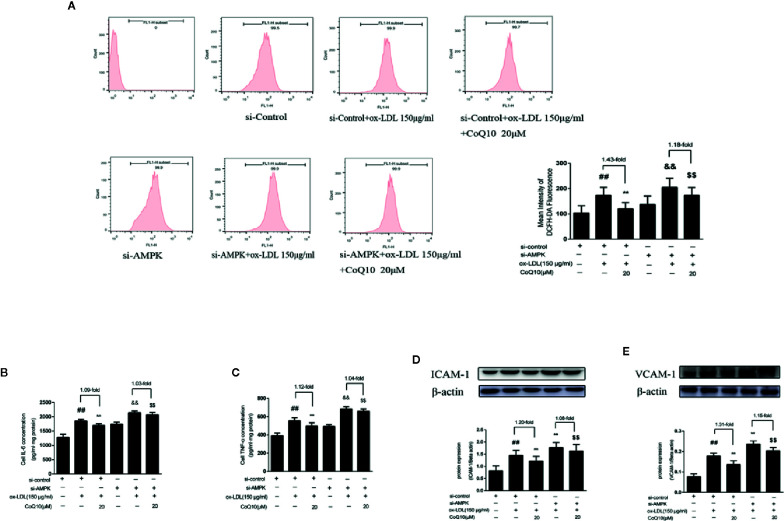
CoQ10 inhibited oxidative stress, reduced ROS production and expression of inflammatory related factors in ox-LDL-induced HAECs by activating AMPK pathway. **(A)** ROS content after si-AMPK. **(B)** ELISA kit results of IL-6 after si-AMPK. **(C)** ELISA Kit results of TNF-α after si-AMPK. **(D)** ICAM-1 protein expression level after si-AMPK. **(E)** VCAM-1 protein expression level after si-AMPK. Data in figure represent the mean ± SD from three independent experiments. ^##^p < 0.01 *vs.* si-Control group, ^**^p < 0.01 *vs.* si-Control + ox-LDL, ^&&^p <0.01 *vs.* si-AMPK group, ^$$^p < 0.01 *vs.* si-AMPK + ox-LDL group.

After AMPK knockdown, we found that the ox-LDL-induced production of ROS was increased in HAECs, and the effect of CoQ10 decreasing ROS production was reduced compared to before AMPK knockdown (**Figure 7A**). At the same time, we detected the related inflammatory indicators. As shown in **Figures 7B, C**, the levels of IL-6 and TNF-α increased significantly after AMPK was knockdown, and the effect of CoQ10 attenuating the levels of IL-6 and TNF-α was inhibited. At the same time, **Figures 7D, E** showed that the protein expression of ICAM-1 and VCAM-1 increased significantly after AMPK knockdown, and the effect of CoQ10 inhibiting ICAM-1 and VCAM-1 was also decreased significantly. Overall, these results proved that AMPK knockdown could enhance oxidative stress in ox-LDL-induced HAECs, increase ROS content, increase related inflammation levels, and weaken the effect of CoQ10, thus accelerating the occurrence of atherosclerosis, suggesting that CoQ10 could prevent against oxidative stress and inflammation by activating AMPK.

### CoQ10 Promotes OPA1 Expression in ox-LDL-Induced HAECs Through YAP Pathway

**Figure 8:**
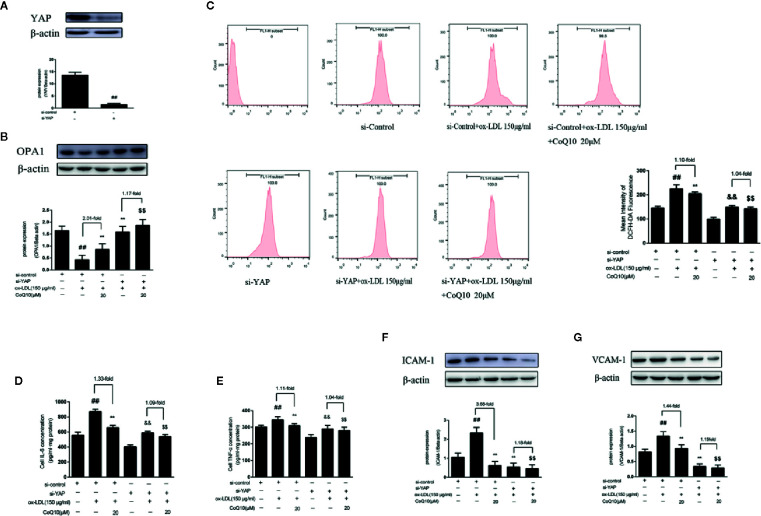
CoQ10 promoted OPA1 expression, inhibited oxidative stress, reduced ROS production and expression of inflammatory related factors in ox-LDL-induced HAECs through YAP pathway. **(A)** YAP protein expression level after transfection of YAP si-RNA. **(B)** OPA1 protein expression level after si-YAP. **(C)** ROS content after si-YAP. **(D)** ELISA kit results of IL-6 after si-YAP. **(E)** ELISA Kit results of TNF-α after si-YAP. **(F)** ICAM-1 protein expression level after si-YAP. **(G)** VCAM-1 protein expression level after si-YAP. Data in figure represent the mean ± SD from three independent experiments. ^##^p < 0.01 *vs.* si-Control group, ^**^p < 0.01 *vs.* si-Control + ox-LDL, ^&&^p < 0.01 *vs.* si-YAP group, ^$$^p < 0.01 *vs.* si-YAP + ox-LDL group.

As shown in **Figure 8A**, the level of YAP protein expression was significantly decreased compared with si-control group, indicating YAP was knockdown successfully. Then, the expression of OPA1 protein after YAP knockout was detected. As seen from **Figure 8B**, the overall trend of OPA1 protein expression was increased after YAP knockdown. However, the effect of CoQ10 up-regulating OPA1 protein expression was decreased compared with that before YAP knockdown. This suggested that CoQ10 might up-regulate the protein expression of OPA1 through YAP pathway.

### CoQ10 Inhibits Oxidative Stress, Reduces ROS Production and Expression of Inflammatory Related Factors in ox-LDL-Induced HAECs by Inhibiting YAP Pathway

After YAP knockdown, we found that the overall trend of ROS production was decreased, and the effect of CoQ10 decreasing ROS production was much more weakened than before YAP knockdown (**Figure 8C**). At the same time, we also detected the related inflammatory indicators. As seen from **Figures 8D, E**, the levels of IL-6 and TNF-α were increased significantly after YAP knockdown, and the effect of CoQ10 decreasing the levels of IL-6 and TNF-α was inhibited. At the same time, **Figures 8F, G** showed that the protein expression of ICAM-1 and VCAM-1was decreased significantly after YAP knockdown, and the effect of CoQ10 reducing the protein expression of ICAM-1 and VCAM-1 was decreased significantly. These results indicated that CoQ10 attenuated oxidative stress by inhibiting YAP pathway and thus reduce ROS production. At the same time, by inhibiting YAP pathway CoQ10 could inhibit expression of the related inflammatory factors, and ultimately protect endothelial cells in atherosclerosis.

## Discussion

There are many theories about the pathogenesis of atherosclerosis. It is generally believed that the occurrence of atherosclerosis is a chronic inflammatory hyperplasia reaction caused by lipid deposition in the subendothelial space due to damage of vascular endothelial cells (Tsai et al., 2012; Larijani et al., 2013). Recent studies have found that atherosclerosis is closely related to aging. At the same time, factors such as inflammation, autophagy damage, mitochondrial dysfunction and excess free radicals more closely influence the development of atherosclerosis at the cellular level (Suárez-Rivero et al., 2019). As a kind of electron transfer, CoQ10 can transfer electrons from mitochondrial respiratory chain compound I (NADH ubiquinone oxidoreductase) and compound II (Ubiquinone succinate oxidoreductase) to the compound III (Ubiquinone cytochrome c reductase). It can play the role of scavenging free radicals and antioxidation in cell membrane and organelle membrane (Yen et al., 2018). It has been reported that CoQ10 supplementation and improvement of mitochondrial function play an important role in the pathophysiology of early atherosclerosis in familial hypercholesterolemia (Suárez-Rivero et al., 2018). Based on these, we found that CoQ10, a mitochondrial energy metabolism enhancer, could produce atherosclerosis attenuating effects by improving mitochondrial energy metabolism in APOE**^−/−^** mice fed with HFD and ox-LDL-induced HAECs.

Coenzyme Q10 is widely used in various diseases due to its high safety and fewer side effects (Belardinelli et al., 2006; Hidaka et al., 2008; Lei and Liu, 2017). In order to explore the clinical efficacy, exogenous supplementation of coenzyme Q10 is usually carried out. Coenzyme Q10 is a fat-soluble substance, and its absorption effect in the body is not ideal. Some studies have reported that it can be improved by repeated continuous administration or changing dosage form (Hyson et al., 2010; Alessio et al., 2019). Related literatures have reported that the doses of coenzyme Q10 were used ranging from 50 to 1,800 mg/kg *in vivo* and the concentrations were used ranging from 2.5 to 100 uM *in vitro* (Tsai et al., 2012; Wang D. et al., 2014; Xu et al., 2017; Zhang et al., 2018; Chen K. et al., 2019; Salehpour et al., 2019). The dose of coenzyme Q10 we used in the *in vivo* experiment is equivalent to 10 mg/kg in human after the calculation of weight conversion coefficient between human and mouse. At the same time, before the *in vitro* experiment, we determined that coenzyme Q10 had almost no toxicity to HAECs within the range of 80 uM by MTT method, so the concentration gradient of 10, 20, 40 uM was set to explore the therapeutic effect of coenzyme Q10.

As an energy sensor, AMPK plays an important role in maintaining energy homeostasis, participating in oxidative stress and inflammatory response (Lee et al., 2012). At the same time, many literatures reported that AMPK can negatively regulate Hippo-YAP pathway and improve mitochondrial function, including increasing OPA1 expression and ATP content (Garrido-Maraver et al., 2015; Wang et al., 2015). In order to further explore the detailed mechanisms of CoQ10 preventing against atherosclerosis by modulating mitochondrial energy metabolism, we validated it *in vitro* and *in vivo*.

*In vivo*, we established the atherosclerosis model by feeding APOE**^−/−^** mice with high fat diet for 12 weeks. It can be observed from the results of oil red O staining and HE staining that CoQ10 had a beneficial effect on the prevention and treatment of atherosclerosis. At the same time, from the results of serum lipid and oxidation levels in mice, we can know that CoQ10 has a good effect in reducing lipid and antioxidation. CoQ10 could also inhibit the inflammatory proteins such as IL-6, TNF-α, ICAM-1, VCAM-1 and NLRP3, thus exerting the role inhibiting atherosclerosis. Then, we detected the expression of proteins associated with energy metabolism. The results showed that CoQ10 could effectively enhance the protein expression of AMPK and OPA1 but inhibit the protein expression of YAP.

Similarly, *in vitro* we used ox-LDL to induce injury in HAECs to establish atherosclerosis model. Relevant indicators were also tested, and the results were basically consistent with those *in vivo*. In order to investigate the effects of CoQ10 on oxidative stress and mitochondrial function induced by ox-LDL, we examined ROS generation, ATP content and mitochondrial membrane potential by JC-1 staining. The results showed that CoQ10 could inhibit oxidative stress, reduce ROS production, improve mitochondrial function and promote energy metabolism.

Furthermore, in order to verify the hypothesis that CoQ10 is associated with AMPK-YAP-OPA1 pathway in the treatment of atherosclerosis. We used gene knockdown technology to explore the mechanism in the present study. When AMPK was knockdown, we found that the phosphorylation of YAP was inhibited, the expression of OPA1 was decreased, and the total content of ATP was decreased. On the contrary, the levels of ROS and the expressions of inflammatory related factors were increased as a whole. More importantly, after AMPK knockdown, the therapeutic effect of CoQ10 was significantly weaker than that before AMPK knockdown. Then we knockdown the YAP gene and detected the related indicators. Similarly, we also found that the therapeutic effect of CoQ10 was significantly reduced after YAP knockdown. This suggested that the protective effect of CoQ10 against atherosclerosis might be related to the AMPK-YAP-OPA1 pathway, and AMPK negatively regulated YAP and promoted the expression of OPA1, thus inhibiting oxidative stress and promoting energy metabolism.

In conclusion, the results *in vivo* and *in vitro* suggest that CoQ10 might improve mitochondrial function, inhibit ROS production and promote energy metabolism by activating AMPK-YAP-OPA1 pathway, thus playing a role in the prevention and treatment of atherosclerosis.

## Data Availability Statement

The raw data supporting the conclusions of this article will be made available by the authors, without undue reservation, to any qualified researcher.

## Ethics Statement

The animal study was reviewed and approved by the institutional animal care committee of Dalian Medical University.

## Author Contributions

HS conceived and designed this study. TX performed major experiments, data analysis, and drafted the manuscript. CW, YJ, QM, QL, and JW provided technological support. All authors contributed to the article and approved the submitted version.

## Funding

The work was supported in part by Grants from the National Natural Science Foundation of China (No. 81273508), and Natural Science Fund of Science and Technology Bureau of Liaoning Province (No. 20180530065).

## Conflict of Interest

The authors declare that the research was conducted in the absence of any commercial or financial relationships that could be construed as a potential conflict of interest.

## Abbreviations

AS, Atherosclerosis; ROS, Reactive oxygen species; CoQ10, Coenzyme Q10; HFD, High fat diet; APOE**^−/−^**, Apolipoprotein E genes knockout; ox-LDL, Oxidized low density lipoprotein; HAECs, Human aortic endothelial cells; siRNA, Small interfering RNA; TG, Triglycerides; TC, Total cholesterol; LDL-c, Low-density lipoprotein cholesterol; HDL-c, High-density lipoprotein cholesterol; LDH, Lactate dehydrogenase; MDA, Malondialdehyde; SOD, Superoxide dismutase; GSH, Glutathione; ATP, Adenosine triphosphate; ICAM-1, Intercellular adhesion molecule 1; VCAM-1, Vascular cell adhesion protein 1; IL-6, Interleukin- 6; TNF-α, Tumor necrosis factor-α; NLRP3, Nucleotide-binding domain leucine-rich-containing family pyrin domain-containing-3; AMPK, Adenosine 5’-monophosphate-activated protein kinase; YAP, Yes-associated protein; OPA1, Optic atrophy 1; DMEM, Dulbecco’s modified eagle medium; FBS, Fetal bovine serum; PBS, Phosphate-buffered saline; EDTA, Ethylenediaminetetraacetic acid; BCA, Bicinchonininc acid; MTT, Methyl thiazolyl tetrazolium; OD, Optical density; DCFH-DA, 2,7-Dichlorodi-hydrofluorescein diacetate; HE, Hematoxylin–eosin staining; PVDF, Polyvinylidene fluoride; TTBS, Tris-buffered saline tween-20; ELISA, Enzyme-linked immuno sorbent assay.
